# Curcumin and Tetrahydrocurcumin as Multi-Organ Modulators of the Adipose Tissue–Gut–Liver Axis: Mechanistic Insights, Therapeutic Potential, and Translational Challenges

**DOI:** 10.3390/ph18121791

**Published:** 2025-11-25

**Authors:** Marina Konaktchieva, Radoslav Stojchevski, Nikola Hadzi-Petrushev, Hristo Gagov, Rositza Konakchieva, Vadim Mitrokhin, Gjoko Kungulovski, Mitko Mladenov, Dimiter Avtanski

**Affiliations:** 1 Gastroenterology Department, Military Medical Academy, Sofia Center, Ul. “Sveti Georgi Sofiyski” 3, 1606 Sofia, Bulgaria; marina.konaktchieva@yahoo.com; 2 Friedman Diabetes Institute, Lenox Hill Hospital, Northwell Health, New York, NY 10022, USA; rstojchevski@northwell.edu; 3 Donald and Barbara Zucker School of Medicine at Hofstra/Northwell, Hempstead, NY 11549, USA; 4 Institute of Molecular Medicine, Feinstein Institutes for Medical Research, Manhasset, NY 11030, USA; 5 Institute of Biology, Faculty of Natural Sciences and Mathematics, Ss. Cyril and Methodius University, 1000 Skopje, North Macedonia; nikola@pmf.ukim.mk (N.H.-P.); djokok@pmf.ukim.mk (G.K.); mitkom@pmf.ukim.mk (M.M.); 6 Department of Animal and Human Physiology, Faculty of Biology, Sofia University “St. Kliment Ohridski”, 1164 Sofia, Bulgaria; hgagov@uni-sofia.bg; 7 Department of Cell and Developmental Biology, Faculty of Biology, Sofia University “St. Kliment Ohridski”, 1164 Sofia, Bulgaria; r.konakchieva@biofac.uni-sofia.bg; 8 Department of Fundamental and Applied Physiology, Russian States Medical University, 117997 Moscow, Russia; mitrokhin_vm@rsmu.ru; 9 Institute of Bioelectronic Medicine, Feinstein Institutes for Medical Research, Manhasset, NY 11030, USA

**Keywords:** curcumin, tetrahydrocurcumin, obesity, metabolic syndrome, adipose tissue, inflammation, gut microbiota, liver, multi-organ axis

## Abstract

Obesity and its related disorders, such as type 2 diabetes mellitus (T2DM) and metabolic dysfunction-associated steatotic liver disease (MASLD), represent a worldwide health challenge, which is driven primarily by the dysfunction of the adipose tissue–gut–liver axis. This article compiles mechanistic and translational data on curcumin and its analogs as multi-organ regulators targeting this axis. Curcumin plays a pleiotropic role by modulating adipogenesis, lipid metabolism, inflammation, fibrosis, and thermogenic remodeling in adipose tissue, tailoring gut microbial diversity, gut barrier integrity, and metabolic endotoxemia. Curcumin in the liver attenuates steatosis, oxidation, and fibrosis by inhibiting lipogenesis, increasing β-oxidation, and modulating the NF-κB and TGF-β signal pathways. These actions result in overall systemic insulin sensitivity and energy balance. On the contrary, the clinical application of curcumin is restricted due to its low solubility, instability, and poor bioavailability. New formulations (nanoparticles/liposomes/micelles) together with structurally enhanced analogs such as tetrahydrocurcumin and monocarbonyl analogs (C66, B2BrBC) exhibited superior pharmacokinetic and tissue-targeting properties in preclinical models. Pilot and randomized clinical trials suggest that curcumin supplementation enhances glucose and lipid metabolism, reduces liver fat content, and modulates inflammatory markers; however, results across studies remain heterogeneous. Large, high-quality multicenter trials using rigorously standardized, bioavailable curcumin formulations are still required to reliably establish the efficacy and safety of curcumin in metabolic diseases. Next steps involve comparing curcumin analogs, conducting multi-omics analyses to understand host–microbiota–organ crosstalk, and determining cooperative approaches with lifestyle and pharmacological interventions. Taken together, curcumin and its next-generation derivatives may offer a novel therapeutic approach to intervene in the adipose tissue–gut–liver axis for the treatment of obesity-related metabolic diseases.

## 1. Introduction

Obesity and the associated metabolic complications, including type 2 diabetes mellitus (T2DM), metabolic dysfunction-associated steatotic liver disease (MASLD), and metabolic syndrome (MetS), continue to represent a major health issue of the 21st century. As reported by the World Health Organization (WHO) in 2024, over 1.0 billion adults worldwide were considered obese, more than double that registered in 1990; obesity rates are higher than 40% in many high-income regions and growing rapidly in middle-income countries [1]. Recent meta-analyses have reported that MASLD (formerly known as NAFLD) currently affects 30–38% of the global population, a number that soars to >40% in subjects with obesity or T2DM [2]. Similarly, MetS is estimated to affect 25–30% of adults globally and plays a fundamental role in the escalation of cardiovascular (CVD) and metabolic diseases [3]. The increasing worldwide prevalence of this disorder emphasizes the necessity for multi-organ treatment approaches that counteract the interrelated pathophysiology involving dysfunctional adipose tissue and hepatic steatosis associated with systemic insulin resistance.

Adipose tissue was once thought to be an inert energy reservoir that stored excess calories from the diet [4,5]. However, it is now appreciated as a dynamic endocrine organ with important roles involved in lipid storage, energy balance, and metabolic regulation [6,7]. Obesity triggers hypoxia, endoplasmic reticulum (ER) stress, and inflammatory activation of hypertrophic adipocytes, which leads to cytokines (e.g., tumor necrosis factor (TNF), interleukin-6 (IL-6)) and an altered adipokine profile (increased leptin, decreased adiponectin), resulting in systemic insulin resistance and hepatic lipogenesis [4,5,6,7,8,9]. Meanwhile, gut microbiota dysbiosis (decreased diversity, increased endotoxin-producing Gram-negative bacteria, and altered short-chain fatty acid production) contributes to inflammation by breaching the intestinal barrier, leading to increased gut permeability and the establishment of metabolic endotoxemia [9,10]. A microbial imbalance further exacerbates adipose and hepatic inflammation by increasing the translocation of lipopolysaccharides (LPS). As the metabolic center of this axis, the liver integrates signals from both adipose tissue and the gut. Dyslipidemia, accumulation of liver fat, and impaired hepatic function are believed to initiate the sequence of events that lead to MASLD progression and a further deterioration in systemic metabolic dysregulation, where excessive free fatty acid (FFA) flux from dysfunctional adipose depots, together with the action of gut-derived endotoxins, drive hepatic steatosis, oxidative stress, and fibrogenesis [11,12,13].

Based on this complex pathophysiology, therapeutic strategies focusing on a single organ or pathway are only partially effective. Hence, there is a growing focus on multi-organ modulators that can simultaneously impact adipose tissue biology, gut microbial ecology, and hepatic metabolism [6]. In this regard, curcumin, a polyphenolic compound found in the rhizome of *Curcuma longa*, has shown great promise for its broad-spectrum biological actions, including anti-inflammatory, antioxidant, and anti-fibrotic effects, as well as metabolic regulation [14,15]. Among these, preclinical studies suggest that curcumin reduces adipose tissue inflammation and enhances intestinal barrier integrity, in addition to other proposed effects, such as modulation of the gut microbiota [10,16,17]. Curcumin exerts these actions by regulating specific signaling pathways, including AMP-activated protein kinase (AMPK), nuclear factor kappa-light-chain-enhancer of activated B cells (NF-κB), peroxisome proliferator-activated receptors (PPARs), and sirtuin-1 (SIRT1), demonstrating its ability to act through various molecular mechanisms [18,19,20,21].

However, its poor oral bioavailability, low aqueous solubility, and tissue penetration severely limit its clinical translation [22]. To overcome these drawbacks, a novel class of structurally modified curcumin analogs (e.g., monocarbonyl compounds, including C66 and B2BrBC) and advanced delivery systems (e.g., nanoparticles, liposomes, and micelles) have been developed, demonstrating enhanced stability, bioavailability, and tissue specificity [23,24,25,26,27,28]. As such, these findings warrant large-scale clinical trials in obese populations and support the development of innovations to modulate the adipose tissue–gut–liver axis efficiently, broadening the potential of curcumin and its enhanced derivatives as novel adjunctive therapeutic agents for metabolic disorders related to obesity.

In this review, we summarize the latest evidence regarding curcumin and its analogs as multi-organ modulators of the adipose tissue–gut–liver axis. This evidence will be discussed in the context of mechanistic studies to understand how curcumin affects adipose tissue remodeling, gut microbiota composition, and hepatic metabolism; review the translatability of preclinical and clinical findings; and consider therapeutic implications as well as the promise of the future development of curcumin analogs with novel delivery strategies for targeting this axis. In this way, a comprehensive perspective is provided for considering curcumin interventions, which we contend is warranted to increase the recognition of its pleiotropic benefits and potential utility across obesity, metabolic syndrome (MetS), and MASLD.

### Methodology

This review used a narrative approach to provide a comprehensive synthesis of the current literature on curcumin and its analogs in metabolic disease. Relevant literature was identified through searches of major scientific databases, including PubMed, Scopus, and Web of Science, covering the period from January 2018 to April 2025. This time frame was selected because previous major reviews have summarized evidence published before 2018. In contrast, numerous mechanistic, formulation, and clinical studies have been published in the past seven years, substantially advancing our understanding of curcumin’s role in metabolic dysfunction and its translational potential. The search strategy included combinations of the following keywords and Boolean operators: “curcumin”, “tetrahydro curcumin”, “curcumin analogs”, “monocarbonyl curcumin”, “obesity”, “metabolic syndrome”, “insulin resistance”, “type 2 diabetes mellitus”, “adipose tissue”, “adipogenesis”, “lipid metabolism”, “gut microbiota”, “intestinal barrier”, “metabolic endotoxemia”, “liver”, “steatosis”, “MASLD”, “inflammation”, “oxidative stress”, “nanoparticle”, “liposome”, “micelle”, “bioavailability”, “clinical trial”, and “randomized controlled trial”. Boolean combinations such as (“curcumin” OR “tetrahydrocurcumin”) AND (“adipose tissue” OR “gut microbiota” OR “liver” OR “metabolic syndrome”) were applied to ensure comprehensive coverage of both preclinical and clinical evidence relevant to the adipose tissue–gut–liver axis. Additional articles were identified through manual searches of reference lists and recent review articles.

Studies were selected based on their relevance to the therapeutic role, mechanisms of action, and formulation strategies of curcumin and its analogs in metabolic diseases. Both preclinical (in vitro and animal) and clinical (human) investigations were included to provide a comprehensive understanding of curcumin’s translational potential. Preclinical studies were analyzed to describe molecular mechanisms—such as effects on inflammation, adipogenesis, and gut barrier integrity—while clinical trials were used to illustrate translational and therapeutic relevance. The inclusion of both types of studies allows the reader to connect mechanistic insights with clinical applications. However, the synthesis emphasizes mechanistic and translational trends rather than an exhaustive listing of every experimental result. To improve transparency, this review provides comprehensive tables summarizing curcumin formulations and delivery systems shown to enhance bioavailability and tissue targeting, as well as key clinical studies involving curcumin or nano-curcumin.

A total of approximately 650 articles were initially identified through the database search (PubMed, Scopus, and Web of Science). After screening titles and abstracts, 320 studies were excluded as they were unrelated to curcumin, metabolic diseases, or the adipose tissue–gut–liver axis. Among the remaining 330 articles, 218 were excluded after full-text review due to duplication, lack of mechanistic or metabolic relevance, or focus on non-metabolic conditions (e.g., cancer, neurodegenerative, or inflammatory bowel disease models). Ultimately, 112 studies were included in this review: 31 preclinical (in vitro and animal), 26 clinical (human) investigations, and 55 reviews and other categories. Exclusion criteria encompassed: (i) studies with incomplete or non-validated outcomes, (ii) non-original articles (e.g., commentaries, editorials, or case reports), (iii) formulations not involving curcumin or its analogs, and (iv) research lacking mechanistic, translational, or metabolic endpoints. This narrative synthesis, therefore, focused on the most representative and methodologically sound publications that address the mechanistic and translational roles of curcumin and its derivatives within the adipose tissue–gut–liver axis. The primary outcomes pre-defined for synthesis in this narrative review included: (i) mechanistic outcomes—effects of curcumin and its analogs on inflammation, oxidative stress, adipogenesis, lipid metabolism, and gut barrier integrity; (ii) translational outcomes—improvements in metabolic parameters (glucose tolerance, insulin sensitivity, lipid profile, hepatic fat content, and systemic inflammatory markers) reported in clinical trials; and (iii) pharmacological outcomes—advancements in formulation, bioavailability, and tissue-targeting properties of curcumin analogs and delivery systems. These outcomes were selected to align with mechanistic and translational evidence and evaluate curcumin’s potential as a multi-organ modulator of the adipose tissue–gut–liver axis.

## 2. Curcumin and Adipose Tissue: Remodeling and Metabolic Reprogramming

### 2.1. Adipose Tissue Dysfunction: From Storage to Endocrine Dysregulation

Adipose tissue expansion in obesity includes both hypertrophy (enlargement of existing adipocytes) and hyperplasia (generation of new adipocytes). In general, hyperplastic growth is associated with preservation of adipocyte function, whereas hypertrophic expansion results in local hypoxia, ER stress, and mechanical compression of the extracellular matrix, creating an inflammatory milieu [8,29]. Hypertrophic adipocytes secrete high levels of pro-inflammatory adipokines, such as TNF, IL-6, resistin, and monocyte chemoattractant protein-1 (MCP-1, *s.* CCL2), while the production of adiponectin, a critical insulin-sensitizing and anti-inflammatory adipokine, is suppressed [5,30]. This secretory change recruits M1-polarized macrophages and other immune cells, promoting local inflammation and systemic insulin resistance. Additionally, adipose tissue fibrosis, driven by TGF-β and characterized by extracellular matrix deposition, contributes to tissue rigidity, reducing lipid storage capacity and promoting ectopic lipid accumulation [31]. Collectively, these processes underscore the pivotal role of adipose tissue dysfunction as a central driver of systemic metabolic imbalance within the adipose tissue–gut–liver axis.

Curcumin, with its multi-target bioactivity, acts as an anti-obesity agent through several mechanisms, including the regulation of adipogenesis, lipid metabolism, inflammation, and fibrosis, making it a potent nutraceutical candidate for obesity [16,32].

### 2.2. Modulation of Adipogenesis and Lipid Metabolism

**Figure 1:**
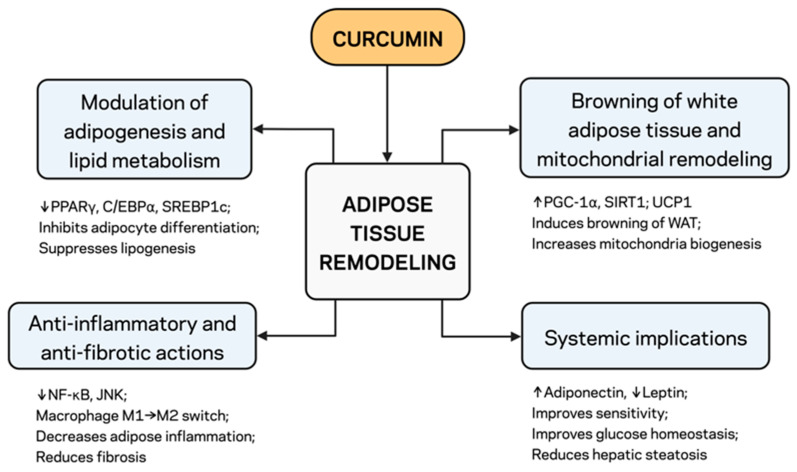
Schematic overview of the adipose tissue–gut–liver axis in obesity, MetS, and MASLD. Curcumin regulates lipid metabolism, inflammation, and insulin sensitivity through the AMPK, PPARγ, and NF-κB pathways. ↓, downregulation; ↑, upregulation. Created with BioRender.com. Stojchevski, R. (2025) https://BioRender.com/8nlkiyk.

Curcumin inhibits adipocyte differentiation through the modulation of transcriptional regulators (Figure 1). It decreases adipogenesis in preadipocytes and downregulates the expression of PPARγ, CCAAT/enhancer-binding protein alpha (C/EBPα), and sterol regulatory element-binding protein 1c (SREBP1c) [20,33]. Curcumin also activates AMPK, the master regulator of energy homeostasis, which in turn enhances fatty acid oxidation and inhibits Acetyl-CoA carboxylase (ACC) and fatty acid synthase (FAS), thus suppressing de novo lipogenesis [34]. Taken together, these effects pose the adipocyte phenotype towards a favorable metabolic profile with reduced TG storage and increased lipid turnover (Figure 1).

### 2.3. Anti-Inflammatory and Anti-Fibrotic Actions

During obesity, people typically develop a type of low-grade inflammation characterized by the presence of immune cells and the secretion of inflammatory cytokines (Figure 1). Curcumin decreases the recycling of TNF, IL-6, and MCP-1 by inhibiting the NF-κB/c-Jun N-terminal kinase (JNK) pathway activation [35]. Curcumin also induces a switch from the M1-state to the anti-inflammatory M2-state in adipose-resident macrophages, thereby reinstating immune homeostasis in the adipose microenvironment [16]. Furthermore, it inhibits TGF-β/SMAD signaling, reduces extracellular matrix deposition, and enhances tissue plasticity, reducing adipose tissue fibrosis [34]. The concomitant suppression of adipose tissue lipolysis and enhancement of its lipid storage capacity increase the ability to accommodate additional lipid excesses, ultimately preventing a lipotoxic spillover to ectopic organs (Figure 1).

### 2.4. Browning of White Adipose Tissue and Mitochondrial Remodeling

Recent evidence indicates that curcumin induces browning in white adipose tissue (conversion of white adipocytes to a beige form, contributing to increased mitochondrial density and uncoupling protein 1 (UCP1) expression) [36,37]. This thermogenic reprogramming is associated with upper curcumin-induced activation of peroxisome proliferator-activated receptor gamma coactivator 1-alpha (PGC-1α) and nicotinamide adenine dinucleotide (NAD^+^)-dependent deacetylase SIRT1, two master regulators of mitochondrial biogenesis and oxidative metabolism [38,39]. Curcumin reduced obesity by inhibiting adipogenesis and enhancing mitochondrial oxidative phosphorylation, thereby increasing energy expenditure and caloric output (Figure 1).

### 2.5. Systemic Implications of Adipose Remodeling

Curcumin acts at the molecular level to restore adipokine balance, increasing adiponectin and reducing serum leptin, while also enhancing systemic insulin sensitivity [40]. Furthermore, curcumin enhances lipid buffering capacity, and decreased inflammatory adipokine secretion results in lower hepatic steatosis and improved peripheral tissue insulin sensitivity [41]. Therefore, the regulation of adipose function by curcumin propagates through a specific network, influencing multiple factors and establishing its identity as a multi-target treatment in MetS.

Recent studies emphasize the plasticity of adipose progenitor cells and their immunometabolic cross-talk as key players in shaping adipose tissue reprogramming. Reciprocal dynamics between the different progenitor subpopulations are observed, particularly for PDGFRα^+^ CD9high adipocyte progenitors, such that phenotypic plasticity determines whether fat mass matures via healthy hyperplastic growth or pathological fibrotic hypertrophy [31]. Their local immune environment strongly influences this behavior; pro-inflammatory M1 macrophages and TH1 lymphocytes favor extracellular matrix deposition and reduced insulin sensitivity, while anti-inflammatory M2 macrophages and regulatory T cells support tissue regeneration while maintaining metabolic homeostasis [29,30]. In this context, curcumin and its hydrogenated derivatives (e.g., tetrahydrocurcumin) mediate the progenitor–immune axis by suppressing NF-κB-dependent cytokine production, repolarizing macrophages toward the M2 phenotype, and activating AMPK–SIRT1–PGC-1α signaling [28,39]. These activities reciprocally promote metabolic adipogenic differentiation towards beige/brown-like lineages, suppress fibrosis, and restore the regenerative/metabolic function of AT during prolonged nutrient deposition (as shown in Figure 1).

Collectively, these findings highlight curcumin’s ability to reprogram dysfunctional adipose tissue toward a metabolically active and less inflammatory phenotype. Such remodeling improves local tissue homeostasis and mitigates systemic metabolic disturbances, forming the basis for its integration into therapeutic strategies for obesity-related disorders.

## 3. Curcumin and Gut Microbiota: Microbial and Barrier Effects

### 3.1. Gut Dysbiosis and Barrier Dysfunction: The Gateway to Metabolic Endotoxemia

**Figure 2:**
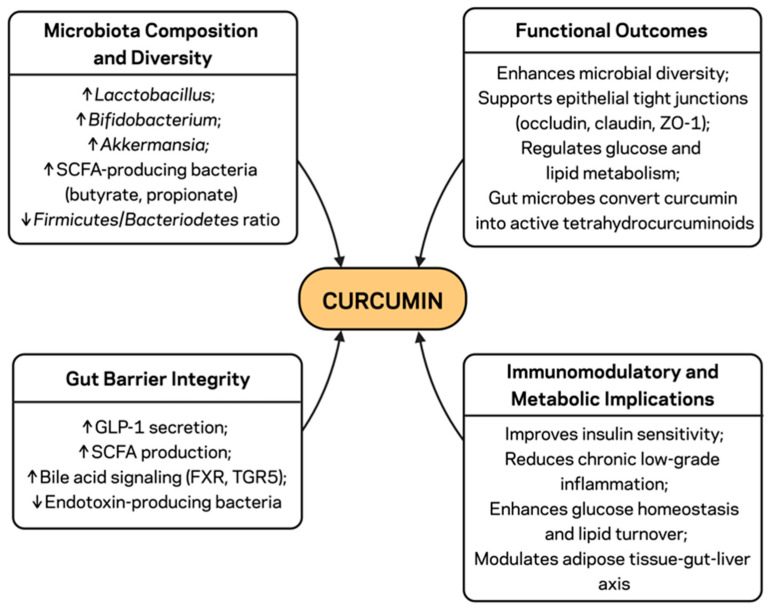
Curcumin’s effects on gut microbiota composition, metabolic activity, and intestinal barrier integrity. Curcumin enhances SCFA-producing bacteria (e.g., *Faecalibacterium*, *Roseburia*, *Bifidobacterium*) and tight-junction proteins (ZO-1, Occludin) while reducing LPS translocation and endotoxemia. Metagenomic and metabolomic analyses indicate curcumin-driven upregulation of butanoate, bile acid, and tryptophan pathways, supporting host–microbial metabolic crosstalk. ↑, an increase; ↓, a decrease. Created with BioRender.com. Stojchevski, R. (2025) https://BioRender.com/rrarn08.

The gut microbiota plays a crucial role in modulating host metabolism by regulating nutrient absorption, bile acid metabolism, and immune homeostasis (Figure 2) [42]. Dietary factors have been shown to modulate the gut microbiota, which may contribute to the development of obesity by increasing the *Firmicutes/Bacteroidetes* ratio, enriching endotoxin-producing Gram-negative bacteria, and depleting short-chain fatty acid (SCFA)-producing species; these changes lead to higher energy uptake from the diet for fat storage [43]. Muscle insulin resistance induced by gut microbiome-driven dysbiosis also leads to loss of tight junction integrity in the intestinal epithelium, causing leaky gut and systemic translocation of microbial endotoxins (a phenomenon known as metabolic endotoxemia) [44]. Increased plasma LPS promotes the activation of Toll-like receptor 4 (TLR4) signaling in adipose tissue and hepatocytes, leading to elevated NF-κB-mediated inflammation and insulin resistance [45]. Dysbiosis, in turn, appears to drive changes in bile acid metabolism and affect farnesoid X receptor (FXR) and Takeda G-protein-coupled receptor 5 (TGR5) signaling, aggravating lipid and glucose homeostasis [46,47].

With its antioxidant and anti-inflammatory properties, curcumin modulates the gut ecosystem to improve barrier integrity and restore microbial homeostasis. These effects might contribute to the systemic metabolic benefits of curcumin, representing a key therapeutic strategy.

### 3.2. Modulation of Microbiota Composition and Diversity

More recent evidence also suggests that curcumin supplementation can beneficially alter gut microbial communities, thereby improving metabolic health. In preclinical studies, an enrichment of potentially beneficial taxa (e.g., *Lactobacillus* spp., *Bifidobacterium* spp., and *Akkermansia* spp.) was associated with enhanced intestinal barrier function and metabolic regulation [48,49]. At the same time, curcumin decreases the rigid *Firmicutes/Bacteroidetes* ratio in obesity [50]. This leads to changes in the primary composition of the gut microbiota and the expansion of SCFA-producing bacteria, which generate butyrate and propionate as key metabolites that support epithelial tight junction function and regulate host glucose and lipid metabolism [51]. In addition, curcumin and microbiota interact bidirectionally: gut microbes, particularly members of the *Bifidobacterium* and *Lactobacillus* genera (Figure 2), facilitate the conversion of curcumin into active metabolites, such as tetrahydrocurcuminoids, which exhibit improved bioavailability and bioactivity [52,53].

### 3.3. Enhancement of Gut Barrier Integrity

Gut dysbiosis associated with obesity disrupts tight junction proteins, leading to increased intestinal permeability and the systemic translocation of LPS [54]. So far, three main tight junction proteins (occludin, claudin-1, and zonula occludens-1 (ZO-1) have been implicated as the therapeutic targets of curcumin to increase the integrity of the gut barrier [55]. This effect prevents LPS leakage into the portal circulation, thereby blunting metabolic endotoxemia and downstream TLR4 activation in adipose tissue and the liver [56]. Recent studies have shown that curcumin lowers circulating LPS and reduces pro-inflammatory cytokine production in rodent models of high-fat diet-induced obesity [57], suggesting that it may be a gut-targeted anti-inflammatory compound.

### 3.4. Immunomodulatory and Metabolic Implications

The beneficial effect of curcumin on modulating gut microbiota has led to systemic metabolic alterations. Additionally, curcumin upregulates glucagon-like peptide-1 (GLP-1) secretion and improves insulin sensitivity by increasing SCFA production [58]. It reduces endotoxin-producing bacteria and enhances barrier-protective effects, thereby improving chronic low-grade inflammation (Figure 2), an essential driver of obesity-associated insulin resistance [59]. Moreover, curcumin can regulate bile acid metabolism and FXR and TGR5 signaling, which impact glucose homeostasis and lipid turnover [60]. Therefore, curcumin affects systemic energy regulation in extra-intestinal sites independently, remodeling the adipose tissue–gut–liver axis (Figure 2).

In addition, beyond compositional control of gut microbiota, recent metagenomic and metabolomic studies have provided new insights into the functional effects of curcumin on microbial activity and its metabolites. Through high-throughput sequencing analyses, it has been shown that curcumin administration increases the levels of SCFA-producing genera, such as *Faecalibacterium prausnitzii*, *Roseburia intestinalis*, and *Bifidobacterium longum* while decreasing pro-inflammatory Gram-negative species, such as *Enterobacteriaceae* and *Desulfovibrio* [49]. Further metabolomic profiling of fecal and plasma samples showed higher levels of butyrate, propionate, and secondary bile acids, suggesting better epithelial barrier integrity and metabolism [54]. In addition, multi-omics integration also identified the modulation of butanoate, tryptophan, and bile acid metabolism as key targets for curcumin to associate these microbial alterations with the promotion of GLP-1 signaling and systemic anti-inflammatory effects [58]. Altogether, these results indicate that curcumin exerts microbiota-targeted effects not only at the compositional level but also at the metabolic and functional levels by modulating host–microbiome interplay (Figure 2).

Next-generation curcumin nanoformulations have been demonstrated to directly alter the structure and function of the gut microbiota. Moreover, multi-omics studies revealed elevated enrichment of Faecalibacterium, Roseburia, and Akkermansia, which boosted butyrate and bile acid production with nano-encapsulated curcumin compared to free curcumin, indicating a significant enhancement in the microbiota-mediated metabolic effects induced by curcumin [49,60].

## 4. Curcumin and the Liver: Lipid Metabolism and MASLD

### 4.1. Hepatic Crosstalk: Convergence of Adipose and Gut-Derived Signals

**Figure 3:**
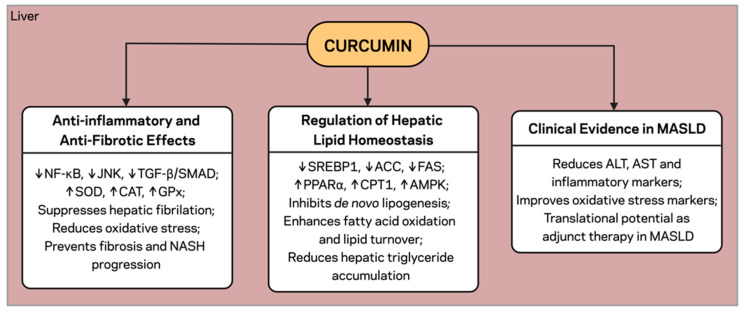
Hepatic effects of curcumin. Curcumin regulates hepatic lipid metabolism by suppressing lipogenesis (SREBP1, ACC, FAS) and enhancing fatty-acid oxidation (PPARα, CPT1, AMPK). It inhibits NF-κB, JNK, and TGF-β/SMAD signaling, increases antioxidant defenses, and improves insulin sensitivity. Clinical studies in MASLD show reductions in hepatic fat, ALT, AST, and inflammatory markers. ↓, downregulation; ↑, upregulation. Created with BioRender.com. Stojchevski, R. (2025) https://BioRender.com/4qossjb.

As a central metabolic hub of the adipose tissue–gut–liver axis, the liver is responsible for carbon partitioning (including lipid and glucose metabolism), detoxification, and maintaining systemic energy homeostasis. A constellation of noxious stimuli converges on the liver during obesity and MetS, including excessive lipid flux (FFA overload from dysfunctional adipose tissue), gut-derived endotoxins, and proinflammatory cytokines (Figure 3), which contribute to the emergence and progression of MASLD [38,61,62,63]. The excessive flux of FFAs from dysfunctional adipose depots exceeds the hepatic capacity for β-oxidation, resulting in triglyceride (TG) storage and lipotoxicity (hepatic steatosis) [11]. At the same time, gut-derived endotoxins and inflammatory mediators activate Kupffer cells and hepatic stellate cells, stimulating NF-κB-mediated inflammation and fibrogenesis [64]. This interplay promotes the transition from simple steatosis to non-alcoholic steatohepatitis (NASH) and advanced fibrosis [65]. Hepatic insulin resistance, further exacerbated by pro-inflammatory cytokines and lipid intermediates, disrupts gluconeogenesis and very-low-density lipoprotein (VLDL) secretion, thereby exacerbating hyperglycemia and dyslipidemia [66]. Therefore, the liver is not only a target but also an amplifier of systemic metabolic perturbations initiated in both adipose tissue and gut.

These inter-organ interactions ultimately establish a vicious cycle that maintains metabolic dysfunction and drives obesity-associated pathologies. Hence, a more recent paradigm is to target this axis rather than through single-organ interventions or drugs [67]. In this regard, bioactive molecules that modulate metabolism across multiple organs simultaneously are expected to exert anti-obesity effects and have potential for treating obesity-associated metabolic disorders. Thus, bioactive compounds, including curcumin, which have pleiotropic effects on inflammation, lipid metabolism, and gut microbial ecology, are attractive candidates for multi-organ metabolic modulation.

Due to its well-described anti-inflammatory, antioxidant, and metabolic-modulating activities, curcumin has been extensively studied in MASLD and MetS models as a hepatoprotective compound that can ameliorate hepatic steatosis, insulin resistance, and fibrogenesis.

### 4.2. Regulation of Hepatic Lipid Homeostasis

An imbalance between lipid acquisition (increased FFA influx and de novo lipogenesis) and disposal (oxidation and export) results in hepatic steatosis (Figure 3). Curcumin has been shown to inhibit de novo lipogenesis by suppressing the expression of SREBP1c and its downstream targets, ACC and FAS [68,69]. On the other hand, curcumin activates PPARα and carnitine palmitoyltransferase 1 (CPT1), thereby facilitating mitochondrial and peroxisomal fatty acid β-oxidation [69]. This dual effect leads to decreased hepatic TG content and increased lipid turnover (Figure 3). In this setting of insulin resistance-driven inflammation, curcumin also helps promote AMPK activation, further enhancing the suppression of lipogenesis and inducing fatty acid oxidation [70], thereby leading to a change in hepatic metabolic characteristics towards a more catabolic and energy-sparing state [71].

### 4.3. Anti-Inflammatory and Anti-Fibrotic Effects

Activation of Kupffer cells and subsequent delivery of gut-derived LPS to the liver continuously initiate hepatic inflammation, leading to NASH development (Figure 3). Curcumin inhibits NF-κB and JNK signaling, thus decreasing the production of pro-inflammatory mediators TNF, IL-6, and MCP-1 [72,73]. It also alleviates oxidative stress by inducing antioxidant enzymes, such as superoxide dismutase (SOD), catalase (CAT), and glutathione peroxidase (GPx), and inhibiting lipid peroxidation [72]. Curcumin lowers hepatic fibrosis by inhibiting TGF-β/SMAD signaling and reduces the activation of hepatic stellate cell-producing extracellular matrix [73]. Taken together, these actions prevent progression from simple steatosis to NASH and advanced fibrosis (Figure 3).

### 4.4. Modulation of Insulin Sensitivity and Glucose Metabolism

A major contributor to the hyperglycemia and dyslipidemia observed in MetS is hepatic insulin resistance. Curcumin improves glucose uptake, insulin receptor substrate-1 (IRS-1) phosphorylation, and protein kinase B (AKT) activity by enhancing the insulin signaling route to stimulate hepatic gluconeogenesis [74]. It corrects hepatic glucose handling by increasing insulin sensitivity and indirectly improves adipose tissue and skeletal muscle metabolism; thus, its benefits are systemic.

### 4.5. Clinical Evidence in MASLD

Clinical trials involving curcumin treatment in MASLD patients showed that hepatic fat fraction and blood transaminases (alanine aminotransferase (ALT) and aspartate aminotransferase (AST)), as well as specific inflammatory markers, were significantly decreased after curcumin intervention [75,76,77,78]. Curcumin supplementation (typically 500–1000 mg/day for 8–12 weeks) has been shown to improve ultrasonographic liver steatosis and circulating markers of oxidative stress in randomized controlled trials [79]. These data are consistent with preclinical data and suggest that curcumin has translational potential as an adjunct therapy in MASLD (Figure 3).

Together, these data indicate that curcumin exerts pleiotropic effects on liver lipid metabolism, inflammation, and fibrosis, associated with the amelioration of insulin sensitivity. Such actions, along with their impact on adipose tissue and gut microbiota, provide a strong rationale for curcumin as a potential multi-organ approach to address obesity-related liver disease. However, the local hepatic activity of curcumin is limited due to its poor systemic bioavailability. When taken orally, curcumin is rapidly metabolized by gut- and liver-mediated conjugation reactions, resulting in low plasma levels and poor tissue exposure [80]. As a result, its hepatic protection is primarily attributed to its indirect actions, including anti-inflammatory and antioxidant effects and modulation of the gut–liver axis to mitigate endotoxin load and systemic metabolic stress. These pharmacokinetic limitations highlight the importance of formulations with increased bioavailability and synthetic analogs that maintain continuous hepatic levels, and hence, metabolic regulation.

## 5. Tetrahydrocurcumin and Curcumin Analogs: Overcoming Limitations

### 5.1. Bioavailability Challenges of Native Curcumin

The intriguing preclinical and clinical data support curcumin’s therapeutic potential; however, its low bioavailability hampers its successful translation. Native curcumin has poor aqueous solubility, is rapidly metabolized in the intestine and liver (via glucuronidation and sulfation), and does not readily penetrate tissues after oral administration, thus limiting systemic exposure to subtherapeutic concentrations [22,80]. Due to these pharmacokinetic obstacles, considerable attention has been devoted to the synthesis of structurally modified curcumin analogs and to the development of advanced delivery technologies to enhance stability (i.e., increased bioavailability), the absorption profile, and tissue specificity (Table 1).

After oral administration, curcumin undergoes rapid metabolism into inactive conjugates and is extensively excreted, resulting in plasma concentrations in the nanomolar range even at high oral doses. This limits its ability to exert sustained biological effects in peripheral tissues, including adipose depots, the gut, and the liver. Furthermore, curcumin’s instability at physiological pH and low permeability across biological membranes additionally compromise its pharmacological potential [80].

### 5.2. Tetrahydrocurcumin: An Active Metabolite with Enhanced Pharmacokinetics

The hydrogenated metabolite of curcumin, produced by gut microbiota with the involvement of hepatic enzymes, is tetrahydrocurcumin (THC), which has high water solubility and chemical stability and appears to be more absorbable compared to its parent compound, curcumin [81]. Clinical studies on THC have shown that THC is twice as potent as an antioxidant, twice as potent as an anti-inflammatory, and five times more effective at lowering low-density lipoproteins (LDLs) than curcumin [82]. THC also improved adipose tissue insulin sensitivity and reduced hepatic steatosis and systemic inflammation in rodent models of obesity and MASLD [83]. Alternatively, THC enhances AMPK activation and drives PGC-1α-mediated mitochondrial biogenesis, effects that are especially relevant for adipose tissue browning and hepatic lipid oxidation [83]. The results of recent experiments suggest that the adipose tissue–gut–liver axis may be targeted more efficiently by THC than native curcumin, and these data propose THC as a candidate for modulating liver-related endotoxemia (Table 1).

Systemic THC facilitates the comparison of pharmacokinetic studies by contrasting its systemic exposure with that of the parent compound. After 20 consecutive oral doses, THC reached a steady-state plasma concentration (C_max_ ≈ 2.5–3.0 µg/mL; half-life (t_1/2_) ≈ 6–8 h) approximately 5–10 times higher than curcumin under comparable conditions (C_max_ ≈ 0.2–0.5 µg/mL; t_1/2_ ≈ 1–2 h). These variations correspond to a significant 3- to 5-fold increase in AUC, indicating enhanced absorption and metabolic stability [80,81,82,83]. Improved solubility, physiological pH stability, and decreased glucuronidation further increase the oral bioavailability of THC, thereby aiding prolonged systemic retention [80,81,82,83]. As such, THC may have greater in vivo antioxidant and anti-inflammatory potential at lower doses, representing an ideal in vivo pharmacological metabolite and a template for curcumin analog development.

### 5.3. Monocarbonyl Curcumin Analogs: Structure–Activity Optimization

To overcome curcumin’s poor bioavailability, newer compounds have been designed, such as those in which the dienone bridge in the center of curcumin is replaced with a more stable linker to increase chemical stability and resistance to metabolism [84]. Preclinical models have demonstrated that such compounds, for example, monocarbonyl derivatives (C66 [85] and B2BrBC [86]), are more potent than curcumin in this regard. These analogs effectively suppress NF-κB activation, reduce the production of pro-inflammatory cytokines, and attenuate oxidative stress-induced cellular damage compared with other compounds (Table 1), demonstrating greater efficacy in ameliorating insulin resistance and hepatic injury [87]. The structure–activity relationship (SAR) studies indicated improved pharmacokinetics and target engagement, including key signaling pathways in metabolic regulation [88]. Although these analogs show promise for improving pharmacokinetics in preclinical models, direct comparative trials in human populations are scarce. The absence of head-to-head comparisons and the lack of consensus on optimal dosing regimens make it challenging to draw robust conclusions regarding efficacy.

### 5.4. Advanced Delivery Systems for Targeted Multi-Organ Effects

In addition to structural modifications, numerous new drug delivery systems have been developed to improve the bioavailability and tissue-specific targeting of curcumin (Table 1). These advantages include formulations such as liposomal and polymeric nanoparticles, micelles, and phospholipid complexes, which are designed to enhance uptake in the gastrointestinal (GI) canal and improve bio-distribution in metabolically active organs [89,90]. The greater efficacy of curcumin in dissipating hepatic lipid accumulation and inflammation than the free form in nanopreparations was demonstrated in MASLD models [27]. These formulations are designed to enhance the bioactivity of orally delivered curcumin by regulating the gut microbiota composition and intestinal epithelial function through its conjugation to a prebiotic-functionalized bioactive delivery system targeted to the colon. At the same time, the plasma bioavailability of systemically accessible nanoformulations would preferentially affect both adipose and hepatic tissues (Table 1). These next-generation compounds and formulations could unlock long-term therapeutic targets and enhance curcumin’s effectiveness in regulating the adipose–organ–gut–liver axis in obesity and metabolic disorders.

Recent advances in curcumin nanoformulations have increased encapsulation efficiency, improved intestinal absorption, and provided more consistent pharmacokinetics. Novel nanocarriers, that is, PEGylated micelles, polymeric nanoparticles, and lipid–polymer hybrid systems, showed improvements in bioavailability and multi-organ distribution in preclinical models of metabolic disorders and cancer [91,92]. This highlights the growing translational opportunities for nanoformulated curcumin beyond cancer treatments in metabolic and inflammatory disorders.

Building on these preclinical and formulation advances, the following section integrates translational perspectives from clinical trials and evaluates the performance of curcumin and its analogs in human studies for metabolic diseases.

## 6. Translational Evidence: From Mechanistic Studies to Clinical Application

Given the strong preclinical evidence demonstrating multi-organ modulation by curcumin and its analogs, translational studies have increasingly evaluated their clinical relevance in human populations with obesity, metabolic syndrome, and MASLD. Although the field of native curcumin has been thoroughly evaluated in patients with MetS, T2DM, obesity, and MASLD (Table 2), breakthroughs in analog development and drug-delivery technologies are promising.

### 6.1. Clinical Outcomes in MetS, MASLD, and Obesity

Clinical evidence shows that curcumin and its nanopreparations are beneficial in patients with MetS, MASLD, and obesity-related metabolic perturbations. Although trends in the same direction—reductions in glycemic control, lipid metabolism, and systemic inflammation—prevailed, the effect’s intensity depends on formulation, dose, or clinical endpoint. Nano-curcumin formulations in particular seem to bring about metabolic improvements at doses much smaller than usual curcumin.

In MetS, relative to the placebo, randomized controlled trials (RCTs) that administered 80 mg/day of nano-curcumin for 8–12 weeks showed significant reductions in triglycerides, fasting insulin, and HOMA-IR, along with improvements in inflammatory biomarkers [99]. Nano-curcumin also decreased leptin and increased the adiponectin levels in similar cohorts with metabolic comorbidities, indicating positive modulation of adipokine signaling [100]. The elucidated mechanism of action, which involves curcumin-induced AMPK activation, enhanced insulin sensitivity, and the inhibition of metabolic inflammation, supports these results [102].

MASLD has shown similar phenomena. Based on these study results, it appears that curcumin at a dose of 500–1500 mg/day for 8 to 12 weeks may help decrease hepatic steatosis and lower AST and ALT [15,72,77,104], as well as LDL, triglycerides (TG), and uric acid [96]. One study of 80 mg/day nano-curcumin in patients with biopsy-confirmed fibrosis showed reduced liver stiffness and GGT levels, suggestive of antifibrotic effects [98]. These favorable effects on the liver are consistent with meta-analysis data demonstrating decreased hepatic fat content, BMI, and inflammatory markers [105].

The evidence on obesity is scarcer. Only one RCT of obese patients with type 2 diabetes provided evidence that the consumption of 1500 mg/day curcumin (12 months) lowered the atherogenic lipid indices and total cardiometabolic risk [101]. Despite encouraging results, there is still a lack of data on obesity-specific clinical outcomes to determine the most effective formulations or dosing cut-offs.

Taken together, the clinical evidence across MetS, MASLD, and obesity underscores curcumin’s ability to modulate metabolic inflammation and lipid–glucose homeostasis by translating mechanistic pathways into discernible clinical efficacy. However, variability in formulations and limited long-term data suggest that curcumin is better established as an adjunct than as a sole therapy.

### 6.2. Tetrahydrocurcumin and Curcumin Analogs in Human Studies

The poor bioavailability of curcumin suggests a potential advantage in using its reduced metabolite, THC, and other chemically modified analogs designed to improve its pharmacokinetic profile. THC shows approximately 1.5- and 2-times greater oral bioavailability and is also pharmacokinetically more stable in comparative PK studies. In a small pilot RCT in T2D, 8-week THC (300 mg/day) treatment decreased the fasting glucose and HbA1c and increased the antioxidant capacity [106].

Nevertheless, THC and monocarbonyl analogs are distinctly under-investigated compounds clinically, unlike curcumin and nano-curcumin, for which several items were reported among numerous RCTs in Table 2. The existing evidence is limited to small pilot studies without cross-replication or direct comparison with nano-curcumin. A larger, well-controlled study is required to determine whether these analogs are more effective from a metabolic or anti-inflammatory perspective.

### 6.3. Pharmacokinetic and Formulation Perspectives

Pharmacokinetic profiles, dosing regimens, and interactions with conventional pharmacotherapy largely dictate the therapeutic efficacy of curcumin in metabolic disorders. In the RCTs summarized in Table 2, traditional curcumin formulations were used at doses ranging from 500 to 1500 mg/day. Still, nano-curcumin was found to have similar or even greater metabolic effects on glycemic parameters when given at a dose equivalent to 80 mg/day due to enhanced solubility and intestinal absorption. Despite curcumin’s weak inhibitory activity for CYP3A4 and P-glycoprotein, the dosing strategies used in these trials did not led to a clinically significant drug–drug interaction. However, co-administration with antidiabetic agents for glucose or lipid management requires vigilant monitoring for an additive metabolic effect [103].

The use of advanced formulation technologies, such as micellar encapsulation, phospholipid complexes, liposomal platforms, and polymeric nanoparticles, has resulted in a several-fold increase in plasma exposure in preclinical and early clinical studies. The existing data indicate that these systems may have metabolic and hepatoprotective effects, but clinical validation is warranted [97,107].

Novel targeted-delivery modalities also aim to fine-tune organ-directed effects. Delivery systems designed for colonic targeting should affect gut microbiota and endotoxemia, whereas systemically distributed nanocarriers are likely to direct delivery to the liver and adipose tissues. Taken together, these pharmacokinetic and formulation strategies constitute significant translational advances for optimizing curcumin bioavailability in the treatment of metabolic diseases.

### 6.4. Regulatory and Safety Considerations

The global commercialization of curcumin and its analogs is challenged by considerable regulatory variability across regions. Numerous curcuminoid-based nutraceuticals are available on the market, differing widely in purity, particle size, excipients, and delivery technologies such as micelles, phospholipid complexes, or nanoparticles [90,91]. These discrepancies markedly affect the pharmacokinetics and bioavailability, thereby limiting the comparability of clinical results and complicating the establishment of standardized dosing guidelines. In most countries, curcumin-containing preparations are regulated as dietary supplements rather than pharmaceuticals, resulting in limited oversight of their efficacy, consistency, and safety. To ensure reliable therapeutic translation, there is an urgent need for harmonized regulatory frameworks that define minimal quality requirements for chemical characterization, stability testing, and bioavailability testing. Collaborative interaction among academia, industry, and regulatory authorities will be essential to bridge the gap between nutraceutical claims and pharmacological validation.

Despite its widespread use and generally favorable safety profile, recent studies emphasize the necessity of systematic toxicological evaluation, especially at pharmacological doses and with nanoformulated or chemically modified curcumin derivatives. High-dose native curcumin (up to 8 g/day for six weeks) is safe, producing only mild gastrointestinal effects and no clinically significant hepatic or renal toxicity [108]. However, data on novel analogs and advanced formulations remain limited.

Certain monocarbonyl analogs, such as C66 and B2BrBC, demonstrate superior stability and potency in preclinical models but may induce oxidative stress or transient hepatic enzyme elevations at supraphysiological doses (>100 mg/kg in rodents) [109]. Likewise, lipid- or polymer-based nanoparticles can alter hepatic metabolism or accumulate in the reticuloendothelial system, underscoring the importance of thorough toxicokinetic and pharmacodynamic profiling [110]. Human data for THC indicate good tolerability up to 400 mg/day, although the safety of long-term or high-dose administration remains insufficiently characterized [111].

Future clinical and regulatory efforts should prioritize comprehensive safety endpoints—including liver function, oxidative stress, and inflammatory markers—to ensure the translational safety of emerging curcumin analogs and delivery systems.

## 7. Conclusions and Future Directions

The adipose tissue–gut–liver axis plays a pivotal role in orchestrating the network underlying the pathogenesis of obesity, MetS, and MASLD. This dysfunction drives a cycle of metabolic rearrangement by propagating a self-amplifying inflammatory axis with a feed-forward loop and a vicious circle involving adipose tissue, the liver, and the gut. The present review highlights the growing body of literature that positions curcumin and its analogs as pleiotropic modulators that target multiple intersections within this axis.

In adipose tissue, curcumin abrogates inflammation and fibrosis caused by hypertrophy, switches immune phenotypes from pro-inflammatory to immature anti-inflammatory profiles, and increases lipolysis/lipophagy and browning, involving the PGC-1α and AMPK pathways. Curcumin alters the microbiome ecosystem to promote increased production of beneficial SCFA-producing taxa, a reduced *Firmicutes*/*Bacteroidetes* ratio, and improved tight junctions, with consequences for limited metabolic endotoxemia. In the liver, a lack of curcumin results in increased SREBP1c-driven lipogenesis and suppressed β-oxidation via PPARα activation, along with aggravated NF-κB/TGF-β signaling and reduced insulin sensitivity, leading to steatosis and fibrosis. To summarize, these effects reinforce curcumin’s role as a multi-organ metabolic modulator that influences distinct yet interconnected metabolic pathways.

**Figure 4:**
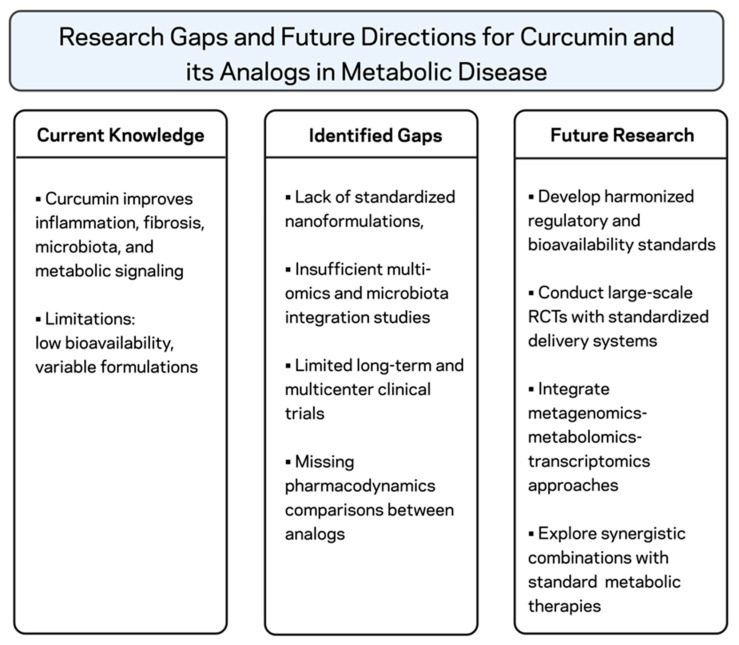
Schematic summary of key research gaps and future directions for curcumin and its derivatives in the management of obesity, MetS, and MASLD. The illustration highlights current evidence, existing limitations, and major research priorities for advancing clinical translation. Created with BioRender.com. Stojchevski, R. (2025) https://BioRender.com/ykfahzm.

Although these mechanistic findings are promising and early clinical trial outcomes appear encouraging, significant obstacles remain—most notably the limited bioavailability of native curcumin, which restricts its therapeutic efficacy (Figure 4). Next-generation delivery strategies are necessary to overcome this significant limitation and fully realize curcumin’s clinical potential. Recent advancements, including the development of THC and monocarbonyl analogs such as C66, B2BrBC, and BrB-COCK-CP35, as well as nanoparticle- or micelle-based delivery platforms, have led to notable improvements in curcumin stability, pharmacokinetics, and tissue bioavailability. Nevertheless, their progression toward broad clinical implementation remains limited. Furthermore, the clinical research landscape is hampered by quick intervention durations, small sample sizes, and heterogeneous formulations and endpoints, thereby preventing definitive conclusions regarding efficacy across patient populations. In addition, the following future research should be prioritized (Figure 4): verification of curcumin efficacy in obesity, MetS, and MASLD by large-scale, multi-center, randomized controlled trials with standardized, bioavailability-enhanced formulations; head-to-head comparisons of curcumin, tetrahydrocurcumin, and their synthetic analogs to identify the best therapeutic candidates; multi-omics analyses (metagenomics, metabolomics, transcriptomics) to gain insight into how curcumin affects host–microbiota cross-talk and inter-tissue communication throughout the adipose–gut–liver axis; characterization of high-dose analogs and advanced delivery systems regarding long-term safety for chronic applications for metabolic diseases, identify synergistic combinations with conventional pharmacotherapies, lifestyle interventions, or other nutraceuticals to enhance therapeutic outcomes.

Curcumin and its analogs constitute a promising class of multi-target therapeutic agents with the potential to modulate the adipose tissue–gut–liver axis, attenuating the severity and preventing obesity-related complications. Continued progress in chemical modification and targeted delivery is positioning curcumin to evolve from a common nutraceutical into a precision metabolic therapy, enabling the rational development of multimodal approaches to prevent and treat challenging metabolic diseases.

## Figures and Tables

**Table 1:** Curcumin formulations and delivery systems have been found to improve bioavailability and tissue targeting. The table summarizes major formulation strategies from preclinical studies developed to overcome the pharmacokinetic limitations of native curcumin. ↑, an increase; ↓, a decrease.

Aspect	Key Features/Mechanism	Functional Outcomes	References
Bioavailability challenges of native curcumin	Poor solubility; rapid metabolism (glucuronidation, sulfation); instability at physiological pH; low membrane permeability	Nanomolar plasma concentrations even at high doses; limited tissue penetration; weak sustained biological activity	[22,78,79,80]
Tetrahydrocurcumin (THC)	Hydrogenated metabolite; ↑ solubility and stability; better absorption; activates AMPK, PGC-1α, mitochondrial biogenesis	Stronger antioxidant and anti-inflammatory vs. curcumin; ↓ LDL; improved insulin sensitivity; ↓ steatosis and systemic inflammation; supports adipose browning and hepatic lipid oxidation; potential to modulate liver-related endotoxemia	[81,82,83,84]
Monocarbonyl analogs (e.g., C66, B2BrBC)	Stable linker replacing dienone bridge; ↑ chemical stability and pharmacokinetics; favorable SAR profile	Potent NF-κB inhibition; ↓ cytokines; ↓ oxidative stress; better efficacy in insulin resistance and hepatic injury; enhanced target engagement in metabolic regulation	[85,86,87,88,89]
Advanced delivery systems	Liposomes, polymeric nanoparticles, micelles, phospholipid complexes; prebiotic-functionalized bioactive carriers	↑ Intestinal absorption and systemic bioavailability; targeted delivery to liver, gut, adipose tissue; improved efficacy in MASLD models; microbiota and epithelial barrier modulation; systemic formulations act on adipose and liver	[90,91,92]

**Table 2:** Clinical Evidence from Randomized Controlled Trials Involving Curcumin/Nano-Curcumin.

Condition/Target	Curcumin Formulations	Sample Size/Duration/Dose	Population	Study Design	Primary Outcomes	Ref.
Prediabetes	Curcumin extract	240/9 months/1.5 g/day	Adults with prediabetes	RCT, double-blind, placebo-controlled	Prevention of T2DM; ↓ HbA1c; ↑ β-cell function	[93]
Type 2 Diabetes	Curcuminoids	100/12 weeks/300 mg/day	Adults with T2DM	RCT, double-blind, placebo-controlled	↓ FFA; ↓ HbA1c; ↓ FPG	[94]
Type 2 Diabetes	Curcuminoids + piperine	100/12 weeks/500 mg/day + 5 mg/day piperine	Adults with T2DM	RCT, double-blind	Improved glycemic control; ↓ ALT/AST; ↓ hs-CRP	[95]
NAFLD/MASLD	Curcumin	80/8 weeks/500 mg/day	Adults with NAFLD	RCT, double-blind	↓ hepatic steatosis; ↓ ALT/AST	[72]
NAFLD/MASLD	Curcumin	102/8 weeks/1000 mg/day	Adults with NAFLD	RCT	↓ LDL; ↓ TG; ↓ uric acid	[96]
NAFLD/MASLD	Curcumin	50/12 weeks/1500 mg/day	Adults with NAFLD	RCT, double-blind	↓ liver inflammation; ↓ cytokines	[77]
NAFLD/MASLD (adjunct)	Phospholipid curcumin	58/8 weeks/250 mg/day	Adults with NAFLD	RCT, double-blind	↓ hepatic fat content; ↓ ALT	[97]
NAFLD fibrosis	Nano-curcumin	55/16 weeks/80 mg/day	Adults with NAFLD + fibrosis	RCT, double-blind	↓ liver stiffness; ↓ GGT; improved fibrosis scores	[98]
Metabolic Syndrome	Nano-curcumin	50/12 weeks/80 mg/day	Adults with MetS	RCT, double-blind	↓ TG; ↓ insulin; improved HOMA-IR	[99]
Migraine/metabolic markers	Nano-curcumin	44/8 weeks/80 mg/day	Adults with migraine	RCT, double-blind	↓ leptin; ↑ adiponectin; ↓ migraine burden	[100]
T2DM + Obesity	Curcumin	227/12 months/1500 mg/day	Overweight/obese adults with T2DM	RCT, double-blind	↓ atherogenic indices; improved metabolic profile	[101]
Metabolic Syndrome	Curcumin extract	65/12 weeks/1890 mg/day	Adults with MetS	RCT, double-blind	↓ TG; ↓ LDL-C; improved non-HDL-C	[40]
Metabolic Syndrome	Curcuminoids + piperine	117/8 weeks/1 g/day + 10 mg/day	Adults with MetS	RCT, double-blind	↑ SOD; ↓ MDA; ↓ CRP	[15]
Type 2 Diabetes	Curcumin	53/10 weeks/1500 mg/day	Adults with T2DM	RCT, double-blind	↓ fasting glucose; ↓ HOMA-IR; ↓ oxidative stress	[41]
Safety/Pharmacokinetics	Curcuminoid formulation	24/3 months/500 mg–12 g/day	Healthy volunteers	Phase I dose escalation	Safety established; Pharmacokinetics characterized	[79]
PK/Bioavailability	Curcumin ± piperine	10/Single dose/2 g ± 20 mg piperine	Healthy volunteers	Randomized cross-over	↑ bioavailability ~20-fold with piperine	[102]
Rheumatoid Arthritis	Bioavailable curcuminoid	24/12 weeks/250–500 mg/day	Adults with Rheumatoid Arthritis	RCT, double-blind	↓ disease activity; ↓ inflammatory markers	[103]

ALT, alanine aminotransferase; AST, aspartate aminotransferase; CRP, C-reactive protein; FFA, free fatty acids; FPG, fasting plasma glucose; GGT, gamma-glutamyl transferase; HbA1c, glycated hemoglobin; HOMA-IR, Homeostatic Model Assessment of Insulin Resistance; LDL, low-density lipoprotein; MDA, malondialdehyde; MetS, metabolic syndrome; NAFLD, non-alcoholic fatty liver disease; MASLD, metabolic dysfunction–associated steatotic liver disease; RCT, randomized controlled trial; SOD, superoxide dismutase; TG, triglycerides; T2DM, type 2 diabetes mellitus; ↓, a decrease or downregulation; ↑, an increase or upregulation.

## Data Availability

No new data were created or analyzed in this study. Data sharing is not applicable to this article.

## Abbreviations

The following abbreviations are used in this manuscript:

ACC	Acetyl-CoA Carboxylase
AKT	Protein Kinase B
ALT	Alanine Aminotransferase
AMPK	AMP-Activated Protein Kinase
AST	Aspartate Aminotransferase
BMI	Body Mass Index
CAT	Catalase
CCAAT	CCAAT/Enhancer-Binding Protein
CD9	Cluster of Differentiation 9
CPT1	Carnitine Palmitoyltransferase 1
CRP	C-Reactive Protein
C/EBPα	CCAAT/Enhancer-Binding Protein Alpha
ER	Endoplasmic Reticulum
FAS	Fatty Acid Synthase
FFA	Free Fatty Acid
FXR	Farnesoid X Receptor
GI	Gastro-Intestinal
GLP-1	Glucagon-Like Peptide 1
GLP-2	Glucagon-Like Peptide 2
GPx	Glutathione Peroxidase
HOMA-IR	Homeostatic Model Assessment of Insulin Resistance
IL-6	Interleukin 6
IR	Insulin Resistance
IRS-1	Insulin Receptor Substrate 1
JNK	c-Jun N-terminal Kinase
LPS	Lipopolysaccharide
MAFLD	Metabolic-Associated Fatty Liver Disease
MASLD	Metabolic Dysfunction-Associated Steatotic Liver Disease
MCP-1	Monocyte Chemoattractant Protein 1
MetS	Metabolic Syndrome
NAD^+^	Nicotinamide Adenine Dinucleotide
NASH	Non-Alcoholic Steatohepatitis
NF-κB	Nuclear Factor Kappa-Light-Chain-Enhancer of Activated B Cells
PDGFRα	Platelet-Derived Growth Factor Receptor Alpha
PGC-1α	Peroxisome Proliferator-Activated Receptor Gamma Coactivator 1-Alpha
PKA	Protein Kinase A
PPARα	Peroxisome Proliferator-Activated Receptor Alpha
PPARγ	Peroxisome Proliferator-Activated Receptor Gamma
SAR	Structure–Activity Relationship
SCFA	Short-Chain Fatty Acid
SIRT1	Sirtuin 1
SOD	Superoxide Dismutase
SREBP1c	Sterol Regulatory Element-Binding Protein 1c
T2DM	Type 2 Diabetes Mellitus
TG	Triglyceride
TGF-β	Transforming Growth Factor-Beta
TGR5	Takeda G Protein-Coupled Receptor 5
THC	Tetrahydrocurcumin
TLR4	Toll-Like Receptor 4
TNF	Tumor-Necrosis Factor
UCP1	Uncoupling Protein 1
VLDL	Very Low-Density Lipoprotein
ZO-1	Zonula Occludens-1
